# Immunohistochemical Analysis of GATA2 Expression in Endometrium and its Relationship with Hormone Receptor Expression in Benign and Premalignant Endometrial Disorders

**DOI:** 10.1007/s43032-024-01730-5

**Published:** 2024-10-23

**Authors:** Aysenur Keske, Usha S. Polaki, Daniel R. Matson

**Affiliations:** Department of Pathology and Laboratory Medicine, University of Wisconsin - Madison, Madison, Wisconsin USA

**Keywords:** GATA2, Progesterone receptor, Endometrium, Endometriosis, Hyperplasia

## Abstract

The GATA gene family encodes highly conserved zinc-finger transcription factors that facilitate the development and function of multiple organ systems including the uterus. In the endometrium, GATA2 functions in a positive autoregulatory loop with the progesterone receptor (PGR) and colocalizes with PGR on chromatin to promote PGR transcriptional programs. GATA2 also has PGR-independent functions that maintain endometrial cell identity, and *GATA2* transcripts reportedly are down-regulated in endometrial disorders including endometriosis. This event is accompanied by a reciprocal increase in *GATA6*. Here, we applied custom anti-GATA2 monoclonal antibodies and performed GATA2 immunohistochemistry (IHC) on patient endometrial tissues corresponding to proliferative, secretory, inactive, and hormone-treated endometrium, as well as endometriosis and endometrial atypical hyperplasia/endometrioid intraepithelial neoplasia (EAH/EIN). We also performed IHC for the estrogen receptor, PGR, and GATA6 in relevant groups. The results reveal a tight correlation between GATA2 and PGR expression in the glandular and stromal cells of benign endometrium. GATA2 expression is markedly reduced in stromal but not glandular cells in endometriosis and EAH/EIN. This reduction in GATA2 expression does not lead to a detectable increase in GATA6 expression in endometriosis. Although average glandular GATA2 expression was preserved in endometriosis and EAH/EIN cases, its expression was decoupled from PGR, implying that alternative pathways regulate GATA2 levels in these disorders. Our findings indicate that GATA2 dysregulation is a feature of endometriosis and EAH/EIN, and support a model whereby loss of stromal GATA2 in these disorders contributes to their progesterone insensitivity.

**Supplementary Information:**

The online version contains supplementary material available at 10.1007/s43032-024-01730-5.

## Introduction

Uterine health and function are regulated by the ovarian steroid hormones estradiol and progesterone, which act on estrogen and progesterone receptors (ERs and PGRs) to mediate critical endometrial activities [1–3]. The endometrium constitutes the functional inner layer of the uterus and is composed of hormone-sensitive glandular and stromal elements (Supplementary Fig. 1). At the start of the menstrual cycle, the endometrium proliferates under the influence of estrogen. Progesterone is secreted by the corpus luteum after ovulation and promotes a secretory transition and endometrial decidualization. These alternating proliferative and secretory phases constitute the endometrial component of the normal menstrual cycle, and result in stereotyped tissue reorganization that can be recognized on routine histologic examination. Following menopause, reduced production of estrogen and progesterone leads to inactive endometrium (absence of normal cycling) and endometrial atrophy [1]. Abnormalities of endometrial hormone receptor activity can compromise endometrial biology and function, and conversely, modulation of hormone receptor activity is leveraged therapeutically to treat endometrial conditions [4, 5].

Unfortunately, disorders of the uterus and endometrium are common and may result in significant symptoms requiring clinical intervention. For example, endometriosis is characterized by abnormal growth of endometrium-like tissue outside of the uterus, frequently involving the ovaries, fallopian tubes, intestines, and other sites, causing pain, infertility, and tissue scarring [5]. Benign endometrial polyps are outgrowths of uterine tissue composed of endometrial glands and stroma. Their size can vary from millimeters up to several centimeters, growth appears to be hormone dependent, and larger pedunculated polyps may interfere with fertility [6]. Finally, endometrial hyperplasia is a disorder in which increased endometrial proliferation is driven by elevated and unopposed estrogen levels [7]. Although usually benign and self-limiting, hyperplastic glands may develop crowding and irregular architecture accompanied by concerning cytologic abnormalities leading to endometrial atypical hyperplasia/endometrioid intraepithelial neoplasia (EAH/EIN), which portends a 22% risk of transformation to endometrioid carcinoma [4].

GATA binding protein 2 (GATA2) is expressed by the endometrium and is one member in a family of six conserved zinc finger transcription factors that bind to GATA nucleotide motifs throughout the genome to promote complex transcriptional programs [8]. GATA2 is a critical regulator of the development and function of the hematopoietic system, prostate, lymphatics, central nervous system, uterus, and other organ systems [9]. Homozygous deletion of *Gata2* in mice causes early embryonic lethality due to failure to form blood, and *Gata2* is critical for maintenance, proliferation, and differentiation of hematopoietic stem and progenitor cells [10]. Patients born with monoallelic mutations in *GATA2* or in enhancers that regulate *GATA2* expression suffer from the GATA2 Deficiency Syndrome, characterized by a constellation of symptoms that show incomplete penetrance and may include bone marrow failure, immunodeficiency, lymphedema, deafness, and others [11, 12]. However, the most devastating outcomes are myelodysplastic neoplasm and acute myeloid leukemia which occur with high penetrance. In addition, 3–5% of apparently sporadic acute myeloid leukemias harbor acquired mutations in *GATA2* [12, 13]. The mechanism(s) by which loss-of-function mutations in *GATA2* and/or reduced GATA2 levels promote myeloid malignancies remains unclear, but the finding has generated interest in targeting GATA2 for the prognosis or treatment of hematologic disorders.

GATA2 also critically regulates endometrial function by driving expression of *PGR* and interacting with PGR on chromatin to reinforce PGR-driven transcriptional programs that are critical for endometrial decidualization and embryo implantation [14, 15]. For this reason, conditional deletion of *Gata2* in the mouse uterus leads to infertility. Loss of *Gata2* is associated with PGR-independent squamous metaplasia of the endometrial lining coupled to a cancer-like gene expression profile, and reduced *GATA2* expression may correlate with aggressive behavior of endometrial carcinomas [16]. In human endometrium, expression of *GATA2* and *PGR* also show a strong positive correlation, while loss of *GATA2* expression may be a defining feature of both adenomyosis and endometriosis, possibly in conjunction with increased expression of *GATA6*, with both GATA family members postulated to play a role in endometriosis pathogenesis [17, 18]. This finding suggests that a pathogenic “GATA switch”, analogous to the GATA2 to GATA1 switch that occurs during normal erythropoiesis, could underlie select endometrial pathologies [19].

Whereas most studies of GATA2 function in the endometrium have focused on measuring transcript levels, it has been more challenging to evaluate the expression level of GATA2 protein in endometrial tissue biopsies from human patients due primarily to a lack of anti-GATA2 antibodies that can routinely detect physiologic levels of GATA2 protein. We recently developed anti-GATA2 monoclonal antibodies which are sensitive and specific for detection of human and mouse GATA2 across multiple assays, including routine immunohistochemistry (IHC) performed on standard formalin-fixed paraffin-embedded (FFPE) pathology specimens [20]. In the present study, we used one of these new antibodies to perform GATA2 IHC on FFPE endometrial biopsies and resections and scored GATA2 expression in endometrial glandular and stromal cells across a cohort of normal endometrium, hormone-treated endometrium, inactive endometrium, endometrial polyps, endometriosis, and EAH/EIN. We also performed IHC for ER, PGR, and GATA6 on relevant case subsets in which a relationship between their expression and GATA2 has been previously reported. In these cases, we scored their expression and analyzed their expression patterns in relation to GATA2. Our results show a strong coupling of GATA2 and PGR expression levels in the stromal and glandular elements of normal endometrium, which is lost in endometriosis and EAH/EIN. We find that while GATA2 expression is lost in the stromal cells of endometriosis and EAH/EIN, we detect no corresponding increase in GATA6. Although overall GATA2 levels are preserved in the glandular cells of endometriosis, GATA2 expression is decoupled from PGR levels, suggesting that PGR-independent pathways regulate GATA2 expression in these cells. Our findings support a model whereby GATA2 dysregulation is a feature of multiple endometrial disorders which can be routinely detected by IHC on patient tissue biopsies.

## Materials and Methods

### IRB and Case Selection

This work was approved by the University of Wisconsin—Madison Institutional Review Board under protocol #2018–1510. All samples used in this study constituted residual material and per protocol patient consent was not required. Cases were identified by searching the electronic medical record at the University of Wisconsin Hospitals and Clinics (UWHC). Pediatric and Veterans Affairs cases were excluded as they were not covered by our IRB protocol. All cases had been previously examined and a diagnosis rendered by a board-certified surgical pathologist with special expertise in gynecologic pathology. The entire case was reviewed again by another board-certified pathologist and the block which best recapitulated the disease process selected for IHC and scoring. Cases with insufficient amounts of glandular and/or stromal elements for analysis were excluded. Except in the hormone treated group, cases in which patients received exogeneous hormone treatment at time of endometrial sampling were excluded.

### Immunohistochemistry

Generation and validation of GATA2 monoclonal antibodies and optimized IHC protocol have been described earlier [20]. IHC for GATA6 (Cell Signaling Technology, Clone D61E4), ER (Ventana, #790–4325), and PGR (Ventana, #790–2223) were performed on the Ventana Discovery Ultra BioMarker platform (Ventana Medical Systems) in a similar fashion. Deparaffinization was accomplished on the instrument followed by heat-induced epitope retrieval with cell conditioner 1 buffer (Ventana #950–224) for 32 min at 95℃. Primary antibodies against ER (Ventana # 790–4325) and PGR (Ventana #790–2223) were pre-diluted and applied to the slide for 60 min (ER) or 32 min (PGR) at 37℃. Primary antibody against GATA6 (Cell Signaling, Ref#5851) was diluted 1:750 with DaVince green diluent (Biocare Medical # PD900H) and applied for 32 min at 37℃. Slides were rinsed with reaction buffer (Ventana #950–300), incubated with Discovery OmniMap anti-rabbit horseradish peroxidase (Ventana #760–4311) for 16 min at 37℃ and then rinsed with reaction buffer. Discovery ChromoMap DAB detection kit (Ventana #760–159) was used for visualization. The slide was then rinsed with warm water and dawn dish soap, and counterstained with Harris hematoxylin (1:5) for 45 s before rinsing, dehydration, dipping in xylene, and coverslipping with mounting media.

### Microscopy and Immunohistochemistry Scoring

Brightfield images were acquired at room temperature on an Olympus BX43 model U-LHLEDC microscope using a 10X air objective with a 0.25 numerical aperture and a Lumenera Infinity 5 camera with Infinity Capture software. All images were white balanced in Adobe Photoshop. Scoring was performed separately by two board-certified pathologists (AK and DRM) and a graduate student (UP). The percent of positive glandular and stromal cells for each marker was evaluated by eye as an average across the entire section. The intensity of marker staining was evaluated on a four-point scale from 0 (negative) to 3 (dark/intense).

### Tissue Culture and Western Blots

ECC1, Ishikawa, and Ark1 cells were cultured in RPMI-1640 + 10% FBS + 1% Penn/Strep. 12z cells were cultured in DMEM/F12 with 10% FBS and 1% Penn/Strep. Sequences for siRNAs are GATA2 Pool #1 (GCACAAUGUUAACAGGCCA, GCGCACAACUACAUGGAAC), GATA2 Pool #2 (GCUUCGAGGAGCUGUCAAA, CCAACAAGUCCAAGAAGAG), and Scramble pool (UAGCGACUAAACACAUCAA, UAAGGCUAUGAAGAGAUAC, AUGUAUUGGCCUGUAUUAG, AUGAACGUGAAUUGCUCAA). Depletions were performed using Lipofectamine RNAiMax (Life Technologies) and 20 nM siRNA according to the manufacturer’s protocol. Depletion efficiency was measured after 48 h by western blotting using the same custom mouse anti-GATA2 monoclonal antibody utilized for IHC at 1:1000 primary concentration. For shRNAs, stable doxycycline-inducible 12z lines were generated by transfecting cells with virions packaged in a pTRIPZ backbone (Horizon Discovery). After transfection, pools were selected for by treatment with 1 µg/mL puromycin and shRNA induced with 1 µg/mL doxycycline was confirmed by IRES-dependent RFP expression. GATA2 depletion was confirmed by western blot. shRNA sequences were shGATA2 #1 (TTCTCTACATAAAGTTGTC) and shGATA2 #2 (TCTTGCTCTTCTTGGACTT).

### Invasion Assays

For Matrigel invasion assays, cells were transferred to Matrigel-coated membrane inserts (membrane 8.0 µm) (Fisher #8774122) 24 h after shRNA induction and allowed to migrate for an additional 24 h, after which Matrigel was removed and the bottom surface of inserts stained with Diff-Quick reagent and imaged by standard microscopy. Cell number was then quantified in a blinded fashion across 5 random fields.

### Statistical Analyses

Student’s T-test was used for between group analyses of two groups. Between group analyses with more than two groups were performed using two-way ANOVA followed by Šidák’s multiple comparisons test. Correlations were calculated using the Pearson method. Curve fitting was accomplished using a variable slope with least squares fit. The threshold for significance was *p* < 0.05 for all analyses.

## Results

### GATA2 IHC in Normal Proliferative and Secretory Endometrium

GATA2 reportedly promotes *PGR* expression under physiologic conditions and works in conjunction with activated PGR to drive PGR-directed transcriptional programs, while also inhibiting estrogen-mediated transcriptional signals [16, 17]. In contrast, *GATA6* is reportedly not expressed at functional levels in normal endometrium [21]. To evaluate the expression of these markers in relation to ER and PGR in human patient samples, we assembled a cohort of 17 proliferative and 19 secretory endometrial biopsies or hysterectomy samples (Table 1) and performed IHC for GATA2 using a custom anti-GATA2 monoclonal antibody that we recently validated [20, 22]. We also performed IHC for ER, PGR, and GATA6. We chose to utilize IHC because it allowed us to directly measure factor protein expression in patient samples while maintaining native tissue architecture, supporting quantification across different cell types. In the case of ER and PGR this was performed using clinically validated antibodies that are routinely used for assessment of these markers in routine surgical pathology practice, while GATA6 IHC was performed using a commercial monoclonal antibody previously utilized for IHC on patient tissue [23, 24]. For all markers, the percent positive cells was scored, as well as staining intensity on a scale from 0 (negative) to 3 (intense).

**Table 1:** Characteristics of endometrial tissue cohort. Immunohistochemistry columns indicates which targets were evaluated for each case cohort

Endometrium Status	Number	Median Age (yrs) [Range]	Immunohistochemistry			
			GATA2	GATA6	ER	PGR
Benign Proliferative	17	38 [24–47]	X	X	X	X
Benign Secretory	19	37 [31–49]	X	X	X	X
Hormone Treated	10	44 [26–58]	X			
Inactive	7	48 [42–78]	X			
Endometrial Polyp	20	49 [31–72]	X			
EAH/EIN	7	60 [35–76]	X		X	X
Endometriosis	18	39 [33–76]	X	X		X

**Fig. 1:**
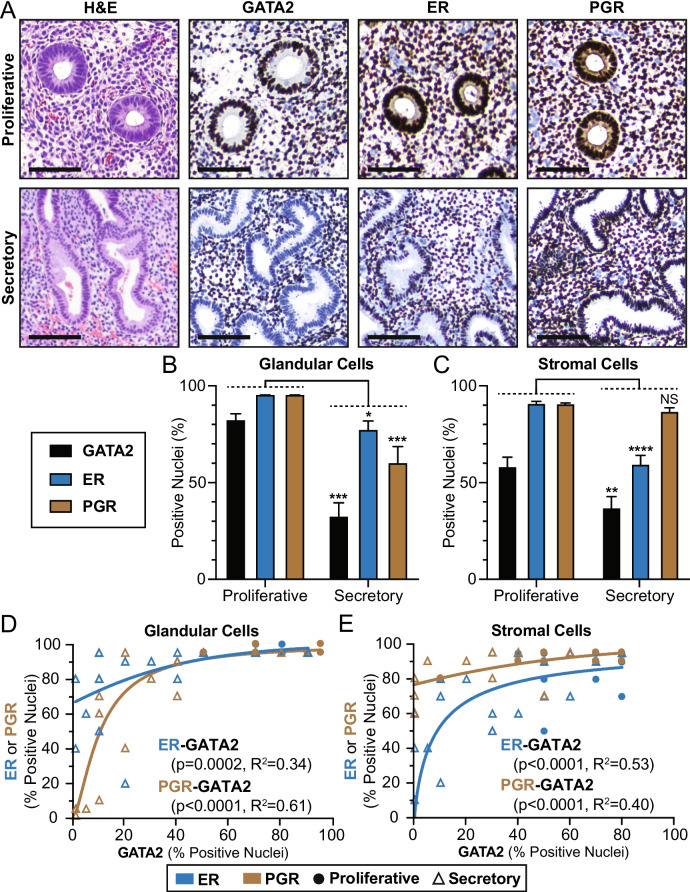
GATA2, ER, and PGR immunohistochemistry in normal cycling endometrium. **A** Representative sections of proliferative and secretory phase endometrium stained for H&E and IHC for GATA2, ER, and PGR. **B-C** Percent GATA2, ER, and PGR positive nuclei in (**B**) glandular and (**C**) stromal cells from proliferative and secretory phase endometrium. Statistical comparisons made within individual markers. **D-E** Correlation between percent cells positive for GATA2 and either ER or PGR in (**D**) glandular and (**E**) stromal endometrial cells. All IHCs are DAB with hematoxylin counterstain. NS = not significant, *p < 0.05, **p < 0.005, ***p < 0.0005, ****p < 0.00005. Scale bar = 50 microns

GATA2 IHC yielded crisp nuclear staining in a subset of glandular and stromal cells that correlated to menstrual phase (Fig. 1A-C). The 83% of glandular cells and 57% of stromal cells that were GATA2-positive (GATA2 +) in proliferative phase endometrium significantly exceeded the 57% of glandular and 34% of stromal cells that were GATA2 + in secretory phase endometrial specimens. The intensity of GATA2 staining also was significantly higher in glandular and stromal cells of proliferative endometrium compared to secretory phase endometrium (Supplemental Fig. 2A-B). Like GATA2, levels of ER and PGR expression were lower in the glandular cells of secretory compared to proliferative phase endometrium (Fig. 1A-B). However, only ER but not PGR expression was significantly reduced in stromal cells (Fig. 1C). Despite this finding, the levels of GATA2, ER and PGR expression were highly correlated in glandular cells and stromal cells in normal endometrium (Fig. 1D-E). As expected, no labeling for GATA6 was identified in the glands or stroma of normal endometrium (Supplemental Fig. 1C). Fallopian tube epithelium showed appropriate nuclear GATA6 labeling and served as a positive control [25].

### GATA2 IHC in Hormone Treated Endometrium, Inactive Endometrium, and Endometrial Polyps

**Fig. 2:**
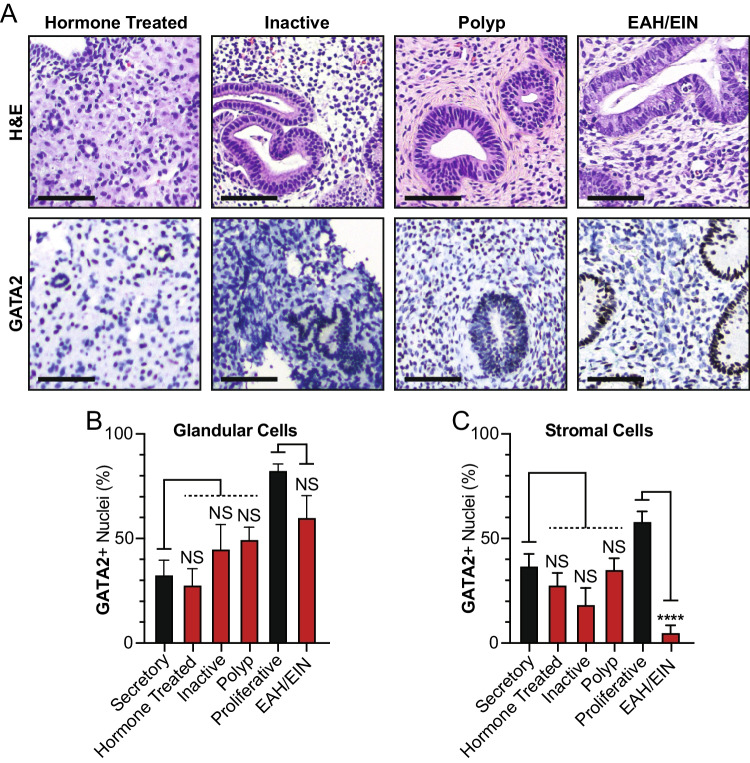
GATA2 immunohistochemistry in hormone treated endometrium, inactive endometrium, endometrial polyps, and endometrial atypical hyperplasia/endometrioid intraepithelial neoplasia. **A** Representative H&E and GATA2 IHC images. **B-C** Percent GATA2 positive nuclei in (**B**) glandular and (**C**) stromal cells. All IHCs are DAB with hematoxylin counterstain. NS = not significant, ****p < 0.00005. Scale bar = 50 microns

GATA2 directly promotes *PGR* expression in the endometrium, and in conjunction with PGR on chromatin it may jointly regulate as many as 50% of endometrial PGR target genes [16]. However, *GATA2* also is a PGR target and endometrial PGR signaling upregulates *GATA2* [15]. This supports a model wherein a positive feedback loop between GATA2 and PGR reinforces cooperative activation of PGR target gene transcription. This model also infers that reduced PGR signaling will result in lower *GATA2* expression. We explored this possibility by performing IHC using anti-GATA2 antibodies on endometrial samples from 9 patients prescribed hormonal birth control comprising either progestin-only (*n* = 5) or combination estrogen and progestin (*n* = 4) regimens. Under both treatment regimens, the endometrium experiences relative progesterone excess, however absence of physiologically elevated estrogen levels also leads to reduced PGR expression, together leading an inactive and decidualized state. First, we confirmed that GATA2 levels in the glands and stroma of endometrium treated with progestin-only regimens was similar to that found in endometrium treated with combination regimens (*p* = 0.88 for glandular cells and *p* = 0.72 for stromal cells). Then, because among the normal endometrial phases, hormone-treated endometrium most closely approximates secretory phase endometrium, we compared GATA2 levels in hormone-treated endometrium against the GATA2 levels we already scored in secretory endometrium (Fig. 1). We found no significant difference between the percent of GATA2-positive cells or in the intensity of GATA2 staining in the glandular or stromal compartments of hormone-treated endometrium compared to normal secretory controls (Fig. 2B-C and Supplementary Fig. 3A-B).

Following menopause, reduced estrogen and progesterone expression by the ovaries compromises ER and PGR endometrial signaling and results in inactive endometrium. We hypothesized that GATA2 expression levels would be reduced in inactive endometrium due to reduced PGR signaling when compared to PGR levels in normal secretory endometrium. However, the percent of glandular and stromal cells positive for GATA2 as well as GATA2 staining intensity in inactive endometrium was similar to normal secretory controls (Fig. 2A-C and Supplemental Fig. 3A-B). Another setting in which endometrial PGR signaling is reduced is in endometrial polyps, which are overgrowths of endometrial glands and stroma that often extend into the uterine cavity [6]. However, we found no difference in GATA2 expression between glands or stroma of endometrial polyps compared to normal secretory endometrium (Fig. 2A-C and Supplemental Fig. 3A-B).

These results suggest that under benign conditions of relatively reduced progesterone receptor activity, GATA2 levels in the glands and stroma are low, and expressed a level similar to normal secretory endometrium.

### GATA2 IHC in Endometrial Atypical Hyperplasia/Endometrioid Intraepithelial Neoplasia

EAH/EIN is a premalignant condition characterized by glandular crowding and cytologic atypia for which exposure to unopposed estrogen is a known risk factor [7, 26]. PGR agonists are often given to patients with simple endometrial hyperplasia and PGR pathways also appear responsive in EAH/EIN considering that these patients often respond to progesterone therapy [27–30]. However, EAH/EIN carries a 22% risk of progression to endometrial carcinoma [31]. Whereas hysterectomy is the definitive treatment for EAH/EIN, continuous progestin-based therapy may be used for poor surgical candidates and young patients who desire fertility sparing therapy [32]. We scored GATA2 and PGR expression in 7 EAH/EIN cases. Because EAH/EIN is a proliferative process, we compared GATA2 expression levels to samples of normal proliferative endometrium that we scored earlier (Fig. 1). We found that GATA2 expression in the glandular compartment of EAH/EIN was similar to normal proliferative endometrium (Fig. 2A-B). However, GATA2 expression in the stromal compartment was markedly reduced, with virtually all EAH/EIN stromal cells showing absence of GATA2 expression (Fig. 2C). A similar pattern of selective GATA2 loss in stroma but not glandular cells of EAH/EIN samples was identified when evaluating intensity of GATA2 and PGR (Supplemental Fig. 3A-B). Moreover, there was no correlation between GATA2 and PGR expression in EAH/EIN, suggesting loss of autoregulation (Supplemental Fig. 3C-D). This was not due to absence of PGR expression, as EAH/EIN cases showed PGR levels similar to normal proliferative endometrium controls (Supplemental Fig. 3E-F).

### GATA2 IHC in Endometriosis

Endometriosis is characterized by implantation of endometrium-like tissue outside of the uterine cavity, often on the ovaries and pelvic surfaces [32, 33]. It arises through still-unclear mechanisms but is associated with both genetic and environmental risk factors [34, 35]. Work in pre-clinical models implicates epigenetic silencing of GATA2 in endometriosis pathogenesis, whereupon loss of *GATA2* expression blunts hormone-dependent transcriptional pathways that contribute to endometriosis pathology [21, 36, 37]. GATA2 loss is reportedly accompanied by upregulation of the related GATA family transcription factor GATA6, at least at the transcriptional level, with elevated *GATA6* blocking hormone responsiveness while simultaneously repressing *GATA2* [21]. However, mechanistic studies have been mostly limited to cultured endometriotic stromal cells, and data on GATA2 protein expression levels (especially in the glandular compartment) in primary endometriotic lesions is lacking.

**Fig. 3:**
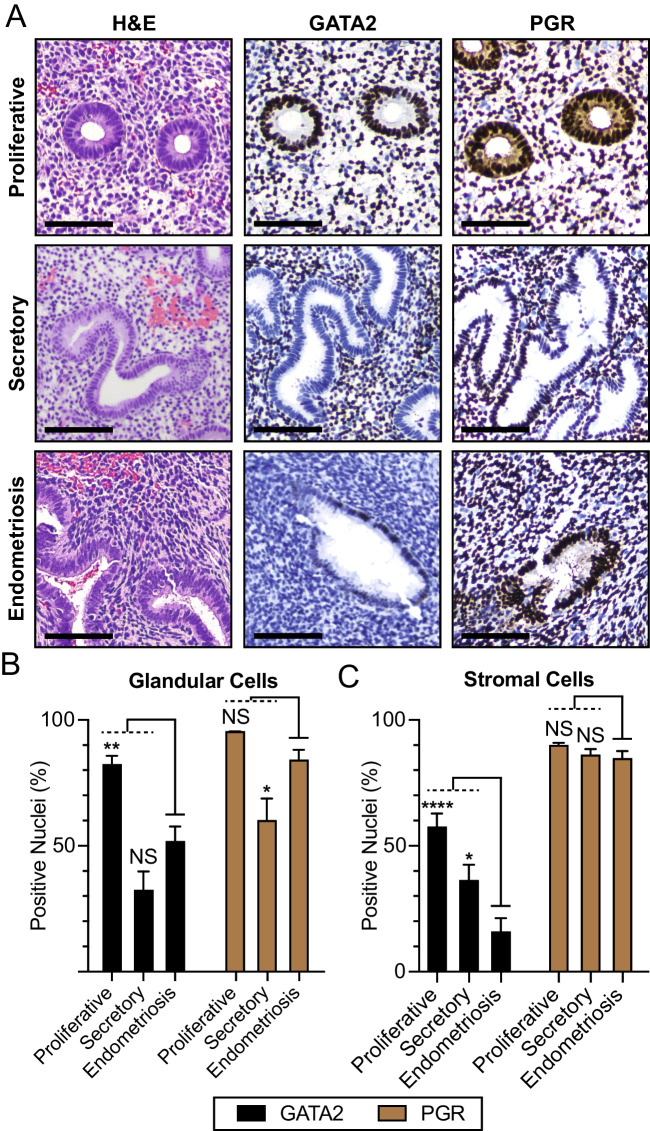
GATA2 and PGR immunohistochemistry in normal cycling endometrium and endometriosis. **A** Representative H&E, and GATA2 and PGR IHC images from normal endometrium in proliferative or secretory phase, and in endometriosis. **B** Percent GATA2 positive nuclei in proliferative and secretory endometrium, and in endometriosis. All IHCs are DAB with hematoxylin counterstain. NS = not significant, *p < 0.05, **p < 0.005, ****p < 0.00005. Scale bar = 50 microns

**Fig. 4:**
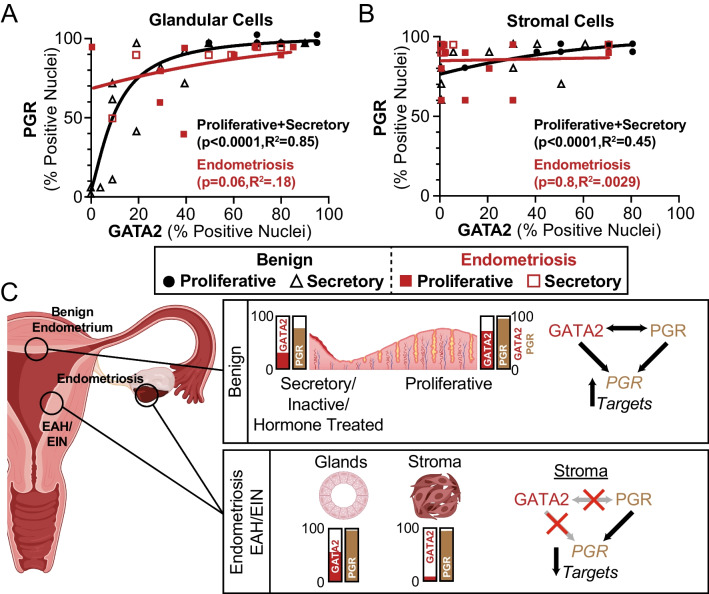
Correlation between GATA2 and PGR IHC expression in normal endometrium and endometriosis. **A-B** Plot of percent nuclei positive for GATA2 and PGR in all analyzed cases of normal endometrium and endometriosis within (**A**) glandular and (**B**) stromal cells. **C** Diagram of GATA2 and PGR expression in normal cycling endometrium contrasted with endometriosis and EAH/EIN. In normal cycling endometrium GATA2 levels are low in secretory phase and high in proliferative phase, while PGR levels are high throughout but highest in proliferative phase. GATA2 levels are lower in inactive and hormone treated endometrium, similar to secretory phrase endometrium. In all conditions, GATA2 remains in a positive feedback loop with PGR while also contributing to PGR gene target transcription. In endometriosis and EAH/EIN, GATA2 levels in stromal cells are markedly reduced, while PGR levels remain high. In stromal cells, GATA2 no longer forms a positive regulatory loop with PGR, and we hypothesize that reduced GATA2 levels fail to contribute to PGR target gene transcription. EAH/EIN = endometrial atypical hyperplasia/endometrioid intraepithelial neoplasia

We scored GATA2, GATA6, and PGR IHC in endometriosis biopsies from 18 patients. The number of GATA2 positive glandular cells in endometriosis lesions was similar to normal secretory endometrium and significantly less than normal proliferating endometrium (Fig. 3A-B). In contrast, glandular PGR expression was elevated to levels measured in proliferative endometrium. However, the number of GATA2-positive stromal cells within endometriotic lesions was significantly reduced below levels measured in normal endometrium regardless of phase (Fig. 3C). This was again in contrast to stromal PGR levels which were no different than normal endometrium. We further correlated the relationship between GATA2 and PGR on a case-by-case basis in endometriosis (Fig. 4A-B). Interestingly, even though glandular GATA2 expression was similar to secretory endometrium controls, GATA2 expression correlated with PGR in benign endometrium whereas this relationship was lost in endometriosis (Fig. 4A). Similarly, there was no significant correlation between GATA2 and PGR expression in the stromal compartment of endometriotic lesions (Fig. 4B). When we stratified benign endometrium and endometriosis cases by menstrual phase, GATA2 expression in the glands and stroma was significantly different in proliferative but not secretory phase (Supplemental Fig. 4). We found virtually no difference in the strength of GATA2 IHC across nuclei (Supplemental Fig. 5A-B), indicating that the number of cells expressing GATA2 reflected the primary driver of differential GATA2 expression across samples. Although earlier studies reported that *GATA6* transcripts are upregulated in endometriotic lesions [21, 38, 39], we found no detectable GATA6 expression in either the glandular or stromal compartments of endometriotic lesions, while background fallopian tube epithelium stained appropriately positive and served as an internal positive control in multiple samples (Supplemental Fig. 5C).

Finally, we sought to determine whether GATA2 depletion may promote extension of endometriosis lesions towards extra-uterine sites. We generated 12z cell lines [40, 41] with doxycycline-inducible expression of anti-GATA2 shRNAs or shScramble controls (Supplemental Fig. 6A). GATA2 depletion in 12z cells did not result in reproducible changes in GATA6 expression (Supplemental Fig. 6B) and we were unable to detect PGR protein or transcripts. We then tested whether GATA2 depletion augmented the ability of 12z cells to invade through Matrigel-coated membranes (Supplemental Fig. 6C). While there was a trend suggesting that GATA2 depletion may increase 12z cell invasion compared to shScramble control, this was not statistically significant (Supplemental Fig. 6D).

## Discussion

Recent studies have established GATA2 as a critical transcription factor in the endometrium that is required for fundamental reproductive functions. An autoregulatory GATA2-PGR axis enables implantation of the fertilized embryo, and GATA2 also supports the fidelity of endometrial tissue identity in a PGR-independent manner. Aberrant *GATA2* expression has been linked to common endometrial disorders including endometriosis. In the current study, our recently validated [42] custom anti-GATA2 monoclonal antibody enabled us to directly evaluate GATA2 protein levels across a range of normal and pathologic patient endometrial samples (partially summarized in Supplemental Table 1).

Our findings in normal cycling endometrium confirm a very strong and positive correlation between GATA2 and PGR expression, which collectively supports the prevailing model in which GATA2 and PGR exist in an autoregulatory positive feedback loop [16]. Our tissue-level analyses extended this paradigm by demonstrating a positive correlation between GATA2 and PGR in both the glandular and stromal compartments of normal cycling human endometrium, and we found no such relationship between GATA2 and ER.

We subsequently evaluated endometrial GATA2 expression in the setting of common benign endometrial conditions associated with abnormal PGR activity including hormone-treated patients, peri/post-menopausal patients who have inactive endometrium, and in the setting of endometrial polyps. Interestingly, we found no significant difference in endometrial GATA2 expression between these conditions and normal secretory phase endometrium. In contrast, we quantified a striking loss of stromal GATA2 expression in EAH/EIN, which was lower than that found in proliferative or secretory phase endometrium. Interestingly, whereas average glandular expression of GATA2 in EAH/EIN appeared to be preserved, further analyses also revealed a decoupling of the GATA2-PGR autoregulatory axis in this cellular compartment as well. We identified the same findings in endometriotic lesions. Our findings support loss of GATA2 and PGR autoregulation in EAH/EIN and endometriosis, and suggest that loss of GATA2 in stromal cells may reliably separate these lesions from normal endometrial tissues (Fig. 4C). It is interesting to speculate that the loss of GATA2 in these lesions may contribute to the progesterone insensitivity that is thought to contribute to disease pathogenesis in both disorders. Maintenance of average GATA2 expression in the glandular cells across these conditions suggests that PGR-independent pathways in this compartment maintain GATA2 at levels indistinguishable from normal endometrium.

Several studies have reported elevated levels of *GATA6* concomitant with loss of *GATA2* in endometriosis [21, 38, 39]. Current models suggest that *GATA2* hypermethylation leads to reduced GATA2 expression in association with *GATA6* hypomethylation. Increased GATA6 expression then both directly represses *GATA2* and independently drives disease pathology, potentially through dysregulation of hormone metabolism [43]. Similar findings have also been reported in adenomyosis, suggesting that a GATA2-GATA6 switch could be a generalizable mechanism across numerous endometrial disorders. This raises the possibility that GATA6 IHC could be worth pursuing as a diagnostic and/or prognostic marker. However, we failed to detect increases in GATA6 expression in endometriotic lesions, even in the presence of appropriately staining internal positive controls. Most prior studies demonstrating elevated *GATA6* in endometriotic tissues have relied on transcript level analyses whereas information related to GATA6 protein expression in endometriosis has been comparatively sparse and sometimes contradictory. Dyson et al. detected elevated GATA6 by immunofluorescence in human cultured endometriotic stromal cells derived from ovarian endometriosis lesions [21]. However, Izawa et al. performed anti-GATA6 IHC on sections of ovarian endometriotic lesions and noted mostly epithelial staining [39]. They also observed that endometriotic lesions from the peritoneum show only marginal anti-GATA6 staining that is localized to glandular cells. Additional tissue-level studies will be required to confirm whether abnormal levels or patterns of GATA6 expression are features of endometriosis and other gynecologic disorders.

Some limitations of our study should be acknowledged. First, interpretation of GATA2, ER, PGR, and GATA6 levels is dependent on antibody sensitivity and specificity, in addition to counterstain properties. The ER and PGR antibodies utilized in our study are clinically validated and utilized in routine diagnostic surgical pathology practice across numerous institutions for the quantitative analysis of ER and PGR levels in human tissues, while our custom anti-GATA2 monoclonal has been validated in human tissues and shows identical staining to a previously-published rabbit polyclonal anti-GATA2 antibody [42, 44]. The monoclonal anti-GATA6 antibody has also been previously utilized for IHC on human tissues [23, 24] and in our hands labeled fallopian tube epithelium appropriately. However, as expression of GATA6 protein in normal and pathologic human endometrium has not yet been systematically interrogated, we cannot rule out that the absence of identifiable GATA6 protein by IHC could be due to an insufficiently sensitive antibody or a function of our IHC staining protocol. Second, all studies seeking to evaluate GATA factor expression must be cognizant of the fact that GATA factor expression levels are often finely regulated in context-specific manners, and levels of individual GATA factors alone may not predict the activity of a given GATA-responsive transcriptional program. Future studies may examine the levels of GATA2 targets in endometrial tissues to determine whether their expression is predicted by GATA2 expression levels measured by IHC. Future studies should also evaluate GATA factor expression in non-human primate models of endometriosis, which represent perhaps the most relevant pre-clinical model for this disorder.

Future studies also may focus on identifying PGR-independent pathways that regulate GATA2 expression in endometrium. In hematopoietic stem and progenitor cells, GATA2 binds to multiple *GATA2* enhancers generating a positive feedback loop and driving further *GATA2* expression. Whether a similar form of GATA2 regulation occurs in the endometrium is not known. It would also be interesting to define GATA2 patterns and expression levels in endometriosis and EAH/EIN following treatment with progestin therapy. Although we and others show that GATA2 levels are suppressed in these disorders, progestin therapy can be effective in treating these entities, suggesting an intact PGR signaling pathway. It may be informative to determine whether progestin therapy leads to upregulation of GATA2 expression, and whether this contributes to PGR transcriptional programs. Interventions which can increase GATA2 expression in these disorders would be expected to increase PGR levels and sensitivity, potentially reducing or ameliorating disease pathology. As EAH/EIN represents a pre-malignant endometrial disorder, and as GATA2 mechanisms are already implicated in malignancies of the prostate and bone marrow, it will also be critical to examine GATA2 levels and activity in endometrial malignancies to determine if GATA2 is also lost in these cancers and whether this contributes to disease pathobiology.

## Supplementary Information

Below is the link to the electronic supplementary material.

Supplemental Figure 1. Anatomy and histology of the uterus and endometrium. (A) Basic anatomy of the uterus and associated structures. The vaginal canal terminates at the cervix, which leads into the uterine cavity. The lining of the uterus from interior to exterior is composed of the endometrium, myometrium, and thin outer serosa. The uterus connects to both fallopian tubes which extend to their fimbriated ends to contact the ovaries. (B) The endometrium composes the functional layer of the uterus, and its cellularity is comprised of both glandular and stromal elements which can be readily appreciated by standard H&E microscopy (PDF 14716 KB)

Supplemental Figure 2. GATA2 and GATA6 IHC in normal cycling endometrium. (A-B) Quantification of GATA2 IHC signal intensity in (A) glandular or (B) stromal cells in proliferative and secretory phase normal endometrium. (C) Representative images of GATA6 IHC from normal cycling endometrium. IHC images are DAB with hematoxylin counterstain. ***p<0.0005 (PDF 1112 KB)

Supplemental Figure 3. GATA2 and PGR IHC in normal cycling endometrium and select endometrial disorders. (A-B) Quantification of GATA2 IHC signal intensity in (A) glandular or (B) stromal cells in proliferative and secretory phase normal endometrium and in select endometrial disorders. (C-D) Correlation between percent cells positive for GATA2 and PGR in the (C) glandular or (D) stromal cells of normal cycling endometrium and in endometrial atypical hyperplasia/endometrioid intraepithelial neoplasia. (E-F) Percent nuclei positive for PGR in benign cycling endometrium and endometrial atypical hyperplasia/endometrioid intraepithelial neoplasia. NS=not significant, ***p<0.0005 (PDF 136 KB)

Supplementary file4 Supplemental Figure 4. GATA2 in normal cycling endometrium and endometriosis stratified by menstrual stage. (A-B) Quantification of GATA2 IHC signal intensity in (A) proliferative or (B) secretory cells in normal cycling endometrium and endometriosis. **p<0.005, ***p<0.0005, NS=not significant (PDF 108 KB)

Supplementary file5 Supplemental Figure 5. GATA2, PGR, and GATA6 IHC in normal cycling endometrium and endometriosis. (A-B) Quantification of GATA2 IHC signal intensity in (A) glandular or (B) stromal cells in normal cycling endometrium and endometriosis. (C) Representative image of GATA6 IHC in endometriosis and fallopian tube epithelium. GATA6 IHC is DAB with hematoxylin counterstain. NS=not significant (PDF 723 KB)

Supplementary file6 Supplemental Figure 6. GATA2 depletion in 12z endometriosis cells does not impact invasion. (A) shScramble and shGATA2 12z cell lines following 48-hour induction with doxycycline or vehicle. (B) GATA6 levels are not consistently different following shGATA2 treatment vs shScramble control. (C) Diagram depicting Matrigel coated membrane invasion assay. After 24 hours doxycycline treatment, 12z cells are loaded into the upper chamber which lacks FBS. Over 24 hours, 12z cells migrate through the Matrigel-coated membrane towards the FBS-containing media in the bottom chamber. (D) There is a trend towards greater 12z invasion after shGATA2 compared to shScramble, but this is not statistically significant. n=11, NS=not significant (PDF 282 KB)

Supplemental Table 1. Summary of GATA2, GATA6, ER, and PGR expression in normal endometrium, endometriosis, and endometrial atypical hyperplasia/endometrioid intraepithelial neoplasia (EAH/EIN) (DOCX 14 KB)

## Data Availability

Primary data is available from the authors upon reasonable request.

## References

[1] Critchley HOD, Maybin JA, Armstrong GM, Williams ARW. Physiology of the endometrium and regulation of menstruation. Physiol Rev. 2020;100:1149–79. 10.1152/physrev.00031.2019. 32031903 10.1152/physrev.00031.2019

[2] Su R-W, Fazleabas AT. Implantation and establishment of pregnancy in human and nonhuman primates. Adv Anat Embryol Cell Biol. 2015;216:189–213. 10.1007/978-3-319-15856-3_10. 26450500 10.1007/978-3-319-15856-3_10 PMC5098399

[3] Fazleabas AT, Strakova Z. Endometrial function: cell specific changes in the uterine environment. Mol Cell Endocrinol. 2002;186:143–7. 10.1016/s0303-7207(01)00655-4. 11900887 10.1016/s0303-7207(01)00655-4

[4] Bińkowska M, Woroń J. Progestogens in menopausal hormone therapy. Prz Menopauzalny. 2015;14:134–43. 10.5114/pm.2015.52154. 26327902 10.5114/pm.2015.52154 PMC4498031

[5] Agarwal N, Subramanian A. Endometriosis – morphology, clinical presentations and molecular pathology. J Lab Physicians. 2010;2:1–9. 10.4103/0974-2727.66699. 21814398 10.4103/0974-2727.66699 PMC3147077

[6] Nijkang NP, Anderson L, Markham R, Manconi F. Endometrial polyps: Pathogenesis, sequelae and treatment. SAGE Open Med. 2019;7:2050312119848247. 10.1177/2050312119848247. 31105939 10.1177/2050312119848247 PMC6501471

[7] Ring KL, Mills AM, Modesitt SC. Endometrial hyperplasia. Obstet Gynecol. 2022;140:1061–75. 10.1097/AOG.0000000000004989. 36357974 10.1097/AOG.0000000000004989

[8] Lentjes MHFM, Niessen HEC, Akiyama Y, de Bruïne AP, Melotte V, van Engeland M. The emerging role of GATA transcription factors in development and disease. Expert Rev Mol Med. 2016;18: e3. 10.1017/erm.2016.2. 26953528 10.1017/erm.2016.2 PMC4836206

[9] Leubolt G, Redondo Monte E, Greif PA. GATA2 mutations in myeloid malignancies: Two zinc fingers in many pies. IUBMB Life. 2020;72:151–8. 10.1002/iub.2204. 31785092 10.1002/iub.2204

[10] Tsai F-Y, Keller G, Kuo FC, Weiss M, Chen J, Rosenblatt M, et al. An early haematopoietic defect in mice lacking the transcription factor GATA-2. Nature. 1994;371:221–6. 10.1038/371221a0. 8078582 10.1038/371221a0

[11] Novakova M, Aliova M, Sukova M, Wlodarski M, Janda A, Froňková E, et al. Loss of B cells and their precursors is the most constant feature of GATA-2 deficiency in childhood myelodysplastic syndrome. Haematologica. 2016;101:707–16. 10.3324/haematol.2015.137711. 27013649 10.3324/haematol.2015.137711 PMC5013954

[12] Wlodarski MW, Hirabayashi S, Pastor V, Starý J, Hasle H, Masetti R, et al. Prevalence, clinical characteristics, and prognosis of GATA2-related myelodysplastic syndromes in children and adolescents. Blood. 2016;127:1387–97. 10.1182/blood-2015-09-669937. 26702063 10.1182/blood-2015-09-669937

[13] Hahn CN, Chong C-E, Carmichael CL, Wilkins EJ, Brautigan PJ, Li X-C, et al. Heritable GATA2 mutations associated with familial myelodysplastic syndrome and acute myeloid leukemia. Nat Genet. 2011;43:1012–7. 10.1038/ng.913. 21892162 10.1038/ng.913 PMC3184204

[14] Rubel C, Jeong J-W, Tsai S, Lydon J, DeMayo F. Epithelial-stromal interaction and progesterone receptors in the mouse uterus. Semin Reprod Med. 2010;28:027–35. 10.1055/s-0029-1242990. 10.1055/s-0029-1242990 20104426

[15] Rubel CA, Franco HL, Jeong J-W, Lydon JP, DeMayo FJ. GATA2 is expressed at critical times in the mouse uterus during pregnancy. Gene Expr Patterns. 2012;12:196–203. 10.1016/j.gep.2012.03.004. 22476030 10.1016/j.gep.2012.03.004

[16] Rubel CA, Wu S-P, Lin L, Wang T, Lanz RB, Li X, et al. A Gata2-dependent transcription network regulates uterine progesterone responsiveness and endometrial function. Cell Rep. 2016;17:1414–25. 10.1016/j.celrep.2016.09.093. 27783953 10.1016/j.celrep.2016.09.093 PMC5084852

[17] Song SH, Jeon MS, Nam JW, Kang JK, Lee YJ, Kang JY, et al. Aberrant GATA2 epigenetic dysregulation induces a GATA2/GATA6 switch in human gastric cancer. Oncogene. 2018;37:993–1004. 10.1038/onc.2017.397. 29106391 10.1038/onc.2017.397

[18] Pavlovic ZJ, Hsin-Yu Pai A, Hsiao T-T, Yen C-F, Alhasan H, Ozmen A, et al. Dysregulated expression of GATA2 and GATA6 transcription factors in adenomyosis: implications for impaired endometrial receptivity. F S Sci. 2024;5:92–103. 10.1016/j.xfss.2023.11.003. 37972693 10.1016/j.xfss.2023.11.003

[19] Bresnick EH, Lee H-Y, Fujiwara T, Johnson KD, Keles S. GATA switches as developmental drivers. J Biol Chem. 2010;285:31087–93. 10.1074/jbc.R110.159079. 20670937 10.1074/jbc.R110.159079 PMC2951181

[20] Robbins DJ, Pavletich TS, Patil AT, Pahopos DC, Lasarev M, Polaki US, et al. Linking GATA2 to myeloid dysplasia and complex cytogenetics in adult myelodysplastic neoplasm and acute myeloid leukemia. Blood Adv. 2023;8(1):80–92. 10.1182/bloodadvances.2023011554. 10.1182/bloodadvances.2023011554 PMC10787255 38029365

[21] Dyson MT, Roqueiro D, Monsivais D, Ercan CM, Pavone ME, Brooks DC, et al. Genome-wide DNA methylation analysis predicts an epigenetic switch for GATA factor expression in endometriosis. PLoS Genet. 2014;10:e1004158. 10.1371/journal.pgen.1004158. 24603652 10.1371/journal.pgen.1004158 PMC3945170

[22] Rajagopalan A, Feng Y, Gayatri MB, Ranheim EA, Klungness T, Matson DR, et al. A gain-of-function p53 mutant synergizes with oncogenic NRAS to promote acute myeloid leukemia in mice. J Clin Invest 2023;133(24). 10.1172/JCI173116. 10.1172/JCI173116 PMC10721149 37847561

[23] Oulès B, Rognoni E, Hoste E, Goss G, Fiehler R, Natsuga K, et al. Mutant Lef1 controls Gata6 in sebaceous gland development and cancer. EMBO J 2019;38:e100526. 10.15252/embj.2018100526. 10.15252/embj.2018100526 PMC6484415 30886049

[24] Oulès B, Deschamps L, Sohier P, Tihy M, Chassac A, Couvelard A, et al. Diagnostic accuracy of GATA6 immunostaining in sebaceous tumors of the skin. Mod Pathol. 2023;36:100101. 10.1016/j.modpat.2023.100101. 36788082 10.1016/j.modpat.2023.100101

[25] Machino H, Dozen A, Konaka M, Komatsu M, Nakamura K, Ikawa N, et al. Integrative analysis reveals early epigenetic alterations in high-grade serous ovarian carcinomas. Exp Mol Med. 2023;55:2205–19. 10.1038/s12276-023-01090-1. 37779141 10.1038/s12276-023-01090-1 PMC10618212

[26] Kurman RJ, Carcangiu ML, Herrington CS, Young RH. WHO classification of tumours of female reproductive organs. 2014.

[27] Ørbo A, Arnes M, Hancke C, Vereide AB, Pettersen I, Larsen K. Treatment results of endometrial hyperplasia after prospective D-score classification: a follow-up study comparing effect of LNG-IUD and oral progestins versus observation only. Gynecol Oncol. 2008;111:68–73. 10.1016/j.ygyno.2008.06.014. 18684496 10.1016/j.ygyno.2008.06.014

[28] Ramirez PT, Frumovitz M, Bodurka DC, Sun CC, Levenback C. Hormonal therapy for the management of grade 1 endometrial adenocarcinoma: a literature review. Gynecol Oncol. 2004;95:133–8. 10.1016/j.ygyno.2004.06.045. 15385122 10.1016/j.ygyno.2004.06.045

[29] Randall TC, Kurman RJ. Progestin treatment of atypical hyperplasia and well-differentiated carcinoma of the endometrium in women under age 40. Obstet Gynecol. 1997;90:434–40. 10.1016/s0029-7844(97)00297-4. 9277658 10.1016/s0029-7844(97)00297-4

[30] Ushijima K, Yahata H, Yoshikawa H, Konishi I, Yasugi T, Saito T, et al. Multicenter phase II study of fertility-sparing treatment with medroxyprogesterone acetate for endometrial carcinoma and atypical hyperplasia in young women. J Clin Oncol. 2007;25:2798–803. 10.1200/JCO.2006.08.8344. 17602085 10.1200/JCO.2006.08.8344

[31] Lacey JV, Ioffe OB, Ronnett BM, Rush BB, Richesson DA, Chatterjee N, et al. Endometrial carcinoma risk among women diagnosed with endometrial hyperplasia: the 34-year experience in a large health plan. Br J Cancer. 2008;98:45–53. 10.1038/sj.bjc.6604102. 18026193 10.1038/sj.bjc.6604102 PMC2359718

[32] Management of endometrial intraepithelial neoplasia or atypical endometrial hyperplasia: ACOG clinical consensus no. 5. Obstet Gynecol 2023;142:735. 10.1097/AOG.0000000000005297. 10.1097/AOG.0000000000005297 37590985

[33] Vercellini P, Viganò P, Somigliana E, Fedele L. Endometriosis: pathogenesis and treatment. Nat Rev Endocrinol. 2014;10:261–75. 10.1038/nrendo.2013.255. 24366116 10.1038/nrendo.2013.255

[34] Smolarz B, Szyłło K, Romanowicz H. Endometriosis: epidemiology, classification, pathogenesis, treatment and genetics (Review of literature). Int J Mol Sci. 2021;22:10554. 10.3390/ijms221910554. 34638893 10.3390/ijms221910554 PMC8508982

[35] Parazzini F, Esposito G, Tozzi L, Noli S, Bianchi S. Epidemiology of endometriosis and its comorbidities. Eur J Obstet Gynecol Reprod Biol. 2017;209:3–7. 10.1016/j.ejogrb.2016.04.021. 27216973 10.1016/j.ejogrb.2016.04.021

[36] Shafrir AL, Farland LV, Shah DK, Harris HR, Kvaskoff M, Zondervan K, et al. Risk for and consequences of endometriosis: A critical epidemiologic review. Best Pract Res Clin Obstet Gynaecol. 2018;51:1–15. 10.1016/j.bpobgyn.2018.06.001. 30017581 10.1016/j.bpobgyn.2018.06.001

[37] Taghizadeh M, Noruzinia M. Lovastatin reduces stemness via epigenetic reprograming of BMP2 and GATA2 in human endometrium and endometriosis. Cell J. 2017;19:50–64. 10.22074/cellj.2016.3894. 28367417 10.22074/cellj.2016.3894 PMC5241518

[38] Yang SC, Park M, Hong K-H, La H, Park C, Wang P, et al. CFP1 governs uterine epigenetic landscapes to intervene in progesterone responses for uterine physiology and suppression of endometriosis. Nat Commun. 2023;14:3220. 10.1038/s41467-023-39008-0. 37270588 10.1038/s41467-023-39008-0 PMC10239508

[39] Izawa M, Taniguchi F, Harada T. GATA6 expression promoted by an active enhancer may become a molecular marker in endometriosis lesions. Am J Reprod Immunol. 2019;81:e13078. 10.1111/aji.13078. 30589192 10.1111/aji.13078

[40] Lee J, Banu SK, Rodriguez R, Starzinski-Powitz A, Arosh JA. Selective blockade of prostaglandin E2 receptors EP2 and EP4 signaling inhibits proliferation of human endometriotic epithelial cells and stromal cells through distinct cell cycle arrest. Fertil Steril. 2010;93:2498–506. 10.1016/j.fertnstert.2010.01.038. 20207353 10.1016/j.fertnstert.2010.01.038

[41] Lee J, Banu SK, Subbarao T, Starzinski-Powitz A, Arosh JA. Selective inhibition of prostaglandin E2 receptors EP2 and EP4 inhibits invasion of human immortalized endometriotic epithelial and stromal cells through suppression of metalloproteinases. Mol Cell Endocrinol. 2011;332:306–13. 10.1016/j.mce.2010.11.022. 21111772 10.1016/j.mce.2010.11.022

[42] Robbins DJ, Pavletich TS, Patil AT, Pahopos D, Lasarev M, Polaki US, et al. Linking GATA2 to myeloid dysplasia and complex cytogenetics in adult myelodysplastic neoplasm and acute myeloid leukemia. Blood Adv. 2024;8:80–92. 10.1182/bloodadvances.2023011554. 38029365 10.1182/bloodadvances.2023011554 PMC10787255

[43] Bernardi LA, Dyson MT, Tokunaga H, Sison C, Oral M, Robins JC, et al. The essential role of GATA6 in the activation of estrogen synthesis in endometriosis. Reprod Sci. 2019;26:60–9. 10.1177/1933719118756751. 29402198 10.1177/1933719118756751 PMC6344952

[44] Im H, Grass JA, Johnson KD, Kim S-I, Boyer ME, Imbalzano AN, et al. Chromatin domain activation via GATA-1 utilization of a small subset of dispersed GATA motifs within a broad chromosomal region. Proc Natl Acad Sci U S A. 2005;102:17065–70. 10.1073/pnas.0506164102. 16286657 10.1073/pnas.0506164102 PMC1287986

