# Supplementary figures and images for: Immunohistochemical Analysis of GATA2 Expression in Endometrium and its Relationship with Hormone Receptor Expression in Benign and Premalignant Endometrial Disorders
Source: Reprod Sci. 2024 Oct 23;31(12):3880–91. doi: 10.1007/s43032-024-01730-5 (PMC11611599; doi:10.1007/s43032-024-01730-5)

**Supplemental Figure 1**

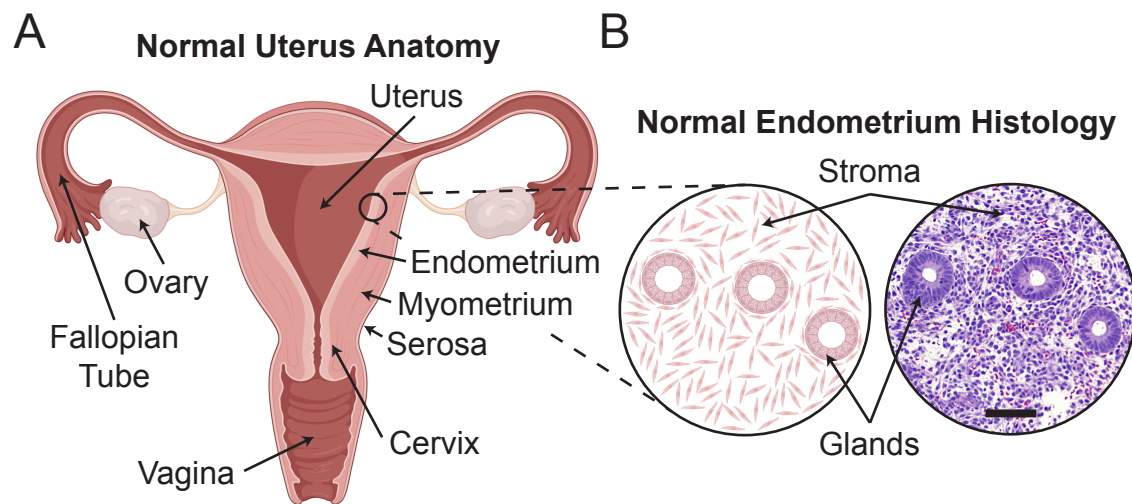

Supplement: Supplementary file 1 — Supplemental Figure 1. Anatomy and histology of the uterus and endometrium. (A) Basic anatomy of the uterus and associated structures. The vaginal canal terminates at the cervix, which leads into the uterine cavity. The lining of the uterus from interior to exterior is composed of the endometrium, myometrium, and thin outer serosa. The uterus connects to both fallopian tubes which extend to their fimbriated ends to contact the ovaries. (B) The endometrium composes the functional layer of the uterus, and its cellularity is comprised of both glandular and stromal elements which can be readily appreciated by standard H&E microscopy (PDF 14716 KB) [file 43032_2024_1730_MOESM1_ESM.pdf]

Supplemental Figure 2

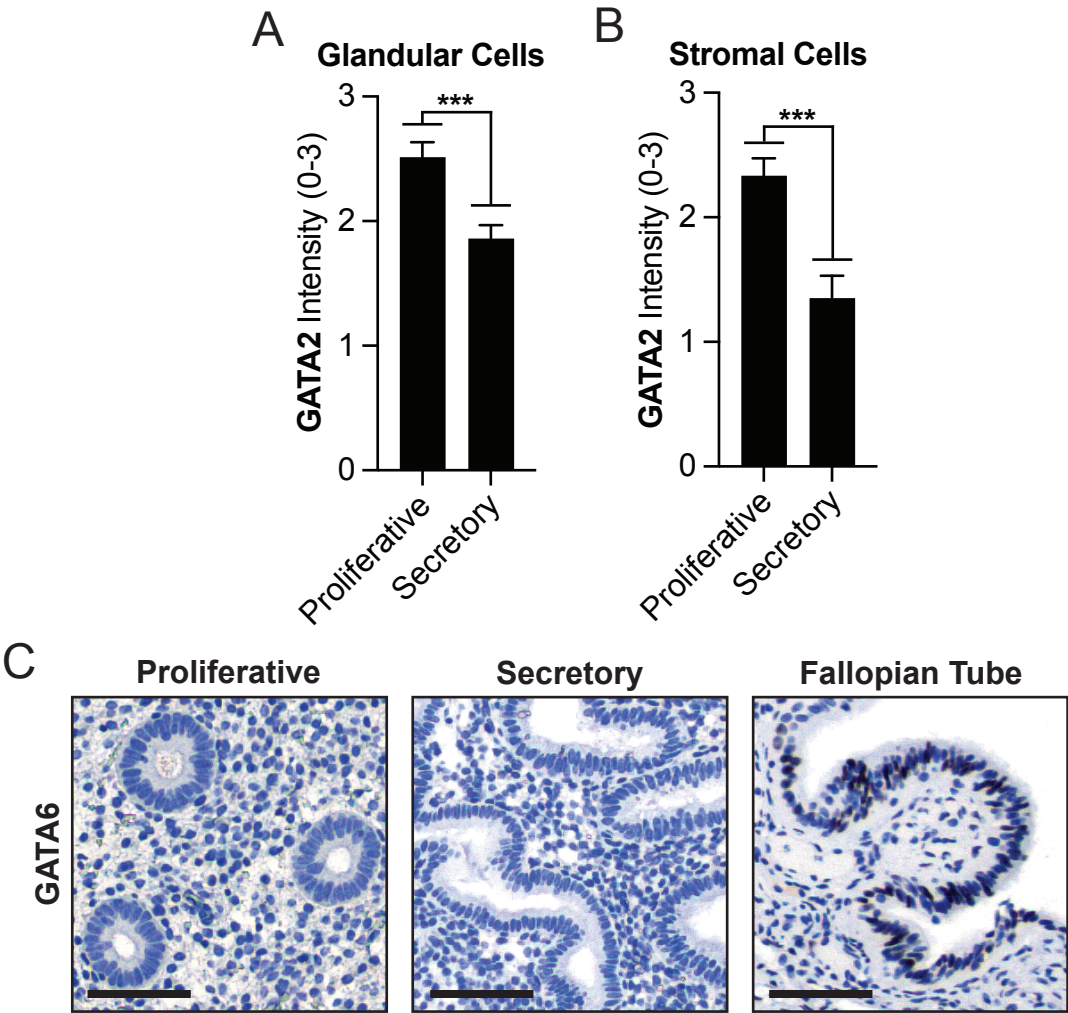

Supplement: Supplementary file 2 — Supplemental Figure 2. GATA2 and GATA6 IHC in normal cycling endometrium. (A-B) Quantification of GATA2 IHC signal intensity in (A) glandular or (B) stromal cells in proliferative and secretory phase normal endometrium. (C) Representative images of GATA6 IHC from normal cycling endometrium. IHC images are DAB with hematoxylin counterstain. ***p<0.0005 (PDF 1112 KB) [file 43032_2024_1730_MOESM2_ESM.pdf]

Supplemental Figure 3

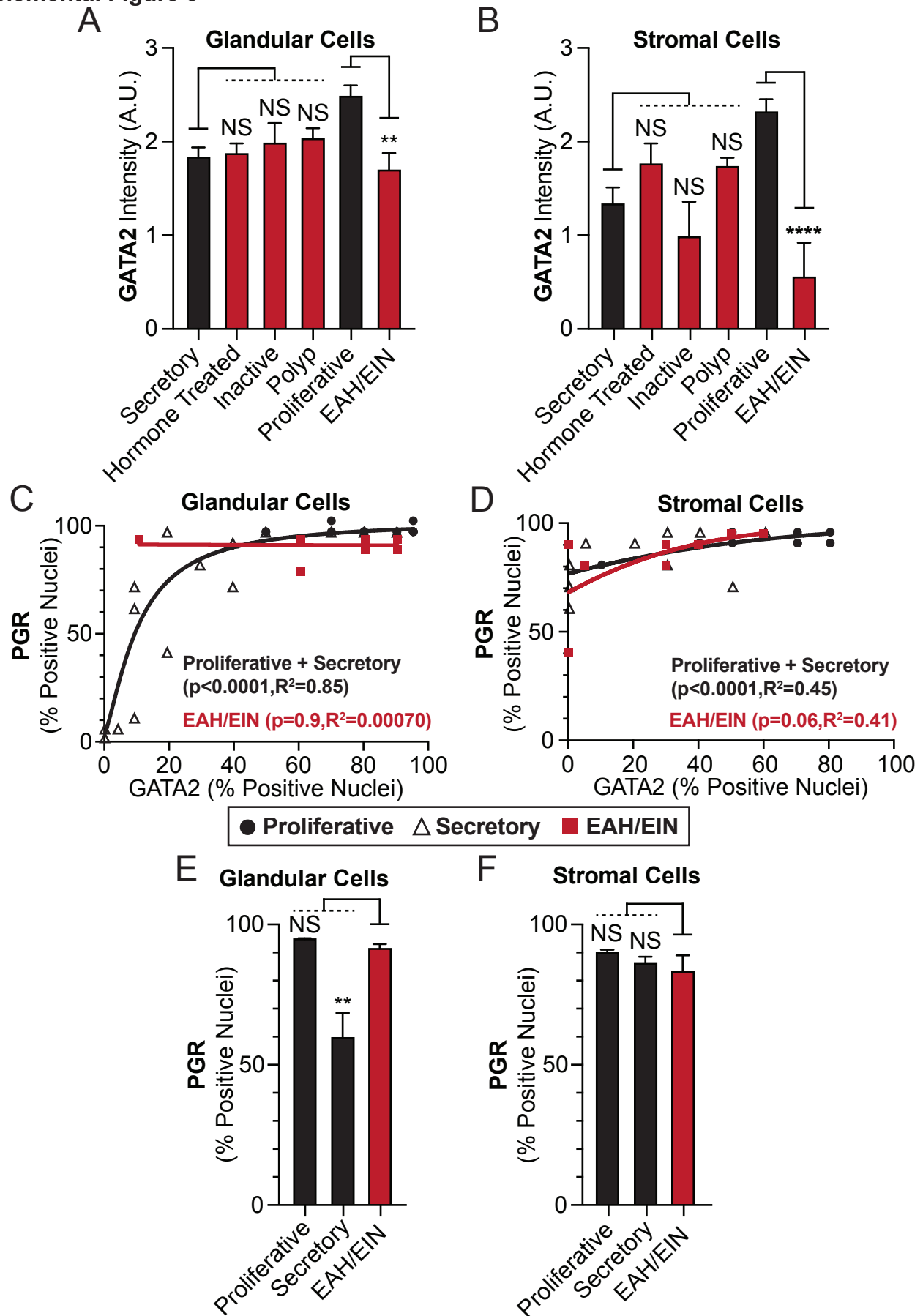

Supplement: Supplementary file 3 — Supplemental Figure 3. GATA2 and PGR IHC in normal cycling endometrium and select endometrial disorders. (A-B) Quantification of GATA2 IHC signal intensity in (A) glandular or (B) stromal cells in proliferative and secretory phase normal endometrium and in select endometrial disorders. (C-D) Correlation between percent cells positive for GATA2 and PGR in the (C) glandular or (D) stromal cells of normal cycling endometrium and in endometrial atypical hyperplasia/endometrioid intraepithelial neoplasia. (E-F) Percent nuclei positive for PGR in benign cycling endometrium and endometrial atypical hyperplasia/endometrioid intraepithelial neoplasia. NS=not significant, ***p<0.0005 (PDF 136 KB) [file 43032_2024_1730_MOESM3_ESM.pdf]

Supplemental Figure 4

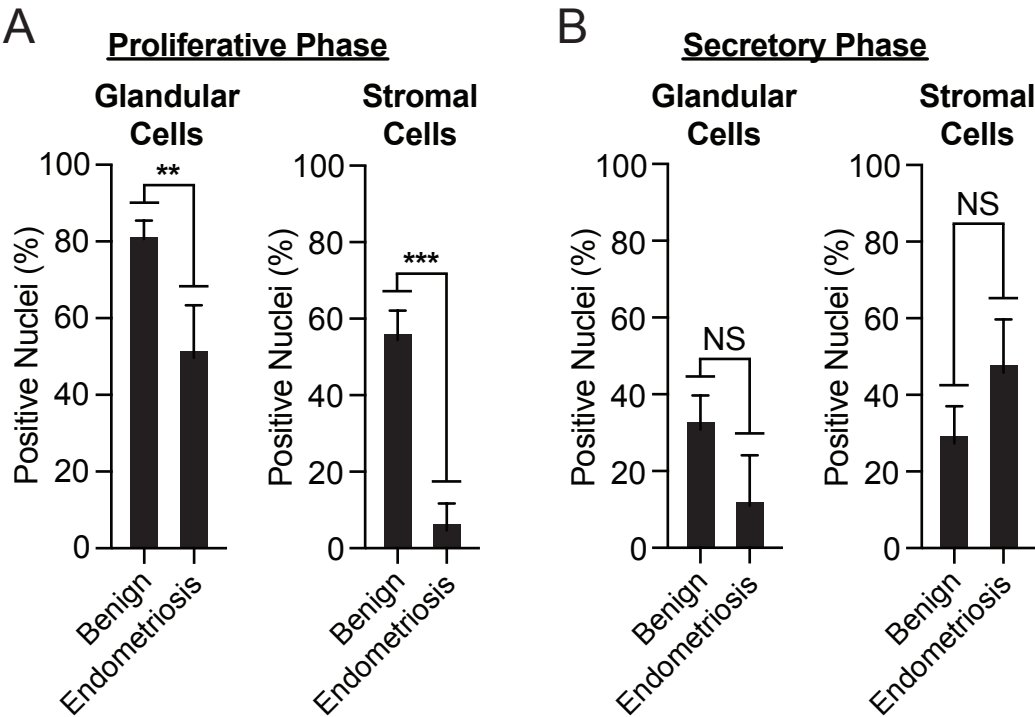

Supplement: Supplementary file 4 — Supplementary file4 Supplemental Figure 4. GATA2 in normal cycling endometrium and endometriosis stratified by menstrual stage. (A-B) Quantification of GATA2 IHC signal intensity in (A) proliferative or (B) secretory cells in normal cycling endometrium and endometriosis. **p<0.005, ***p<0.0005, NS=not significant (PDF 108 KB) [file 43032_2024_1730_MOESM4_ESM.pdf]

Supplemental Figure 5

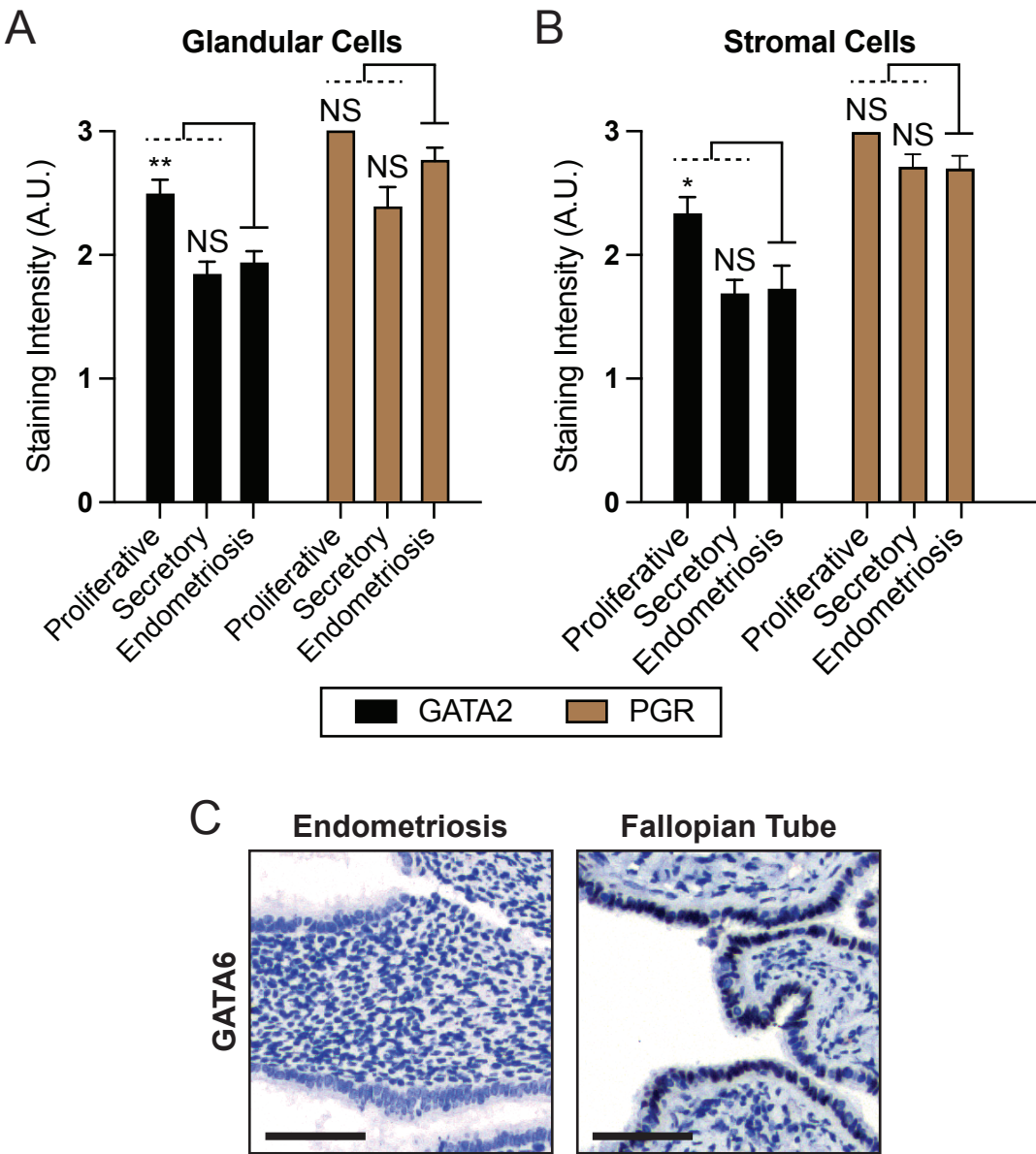

Supplement: Supplementary file 5 — Supplementary file5 Supplemental Figure 5. GATA2, PGR, and GATA6 IHC in normal cycling endometrium and endometriosis. (A-B) Quantification of GATA2 IHC signal intensity in (A) glandular or (B) stromal cells in normal cycling endometrium and endometriosis. (C) Representative image of GATA6 IHC in endometriosis and fallopian tube epithelium. GATA6 IHC is DAB with hematoxylin counterstain. NS=not significant (PDF 723 KB) [file 43032_2024_1730_MOESM5_ESM.pdf]

Supplemental Figure 6

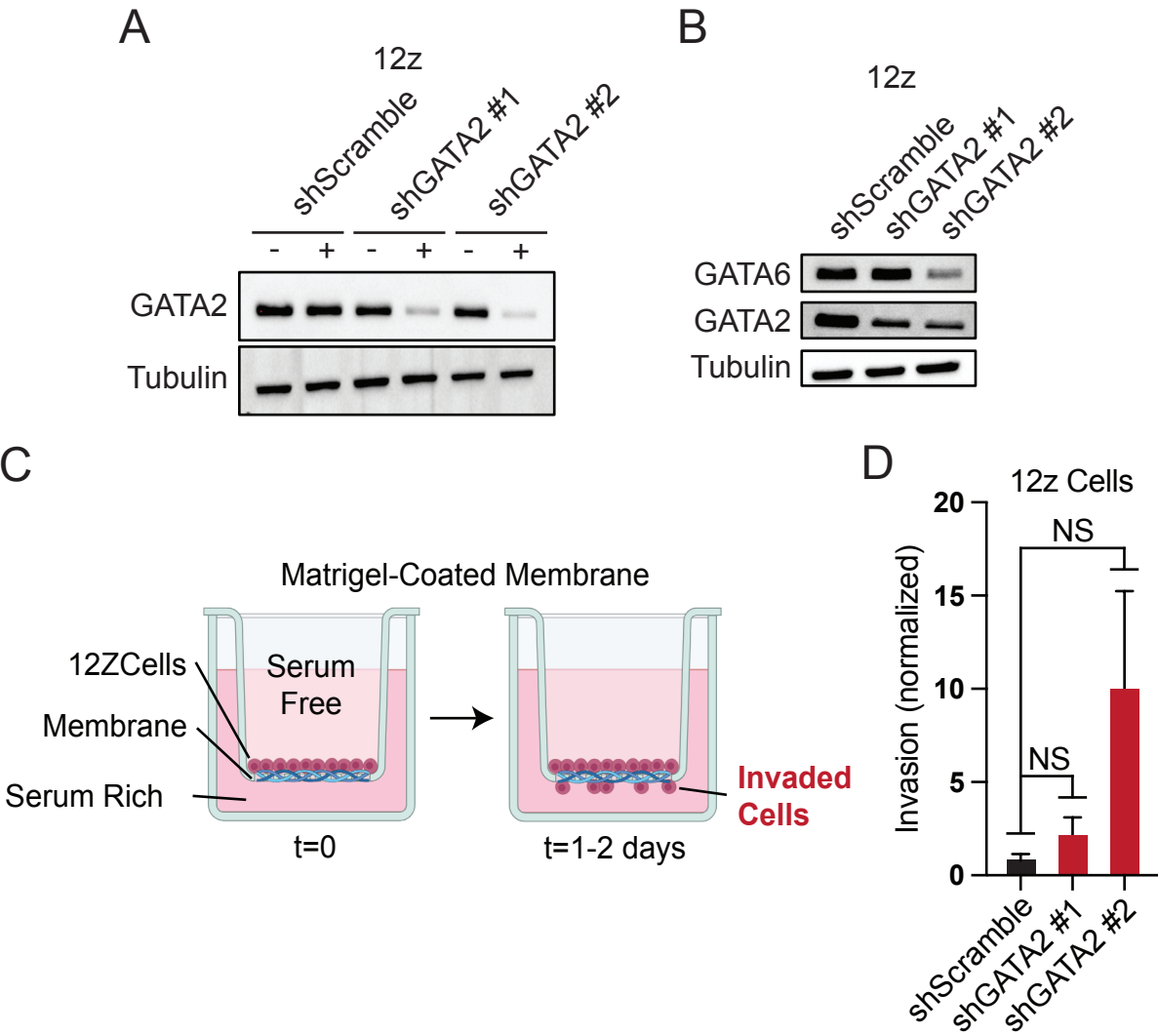

Supplement: Supplementary file 6 — Supplementary file6 Supplemental Figure 6. GATA2 depletion in 12z endometriosis cells does not impact invasion. (A) shScramble and shGATA2 12z cell lines following 48-hour induction with doxycycline or vehicle. (B) GATA6 levels are not consistently different following shGATA2 treatment vs shScramble control. (C) Diagram depicting Matrigel coated membrane invasion assay. After 24 hours doxycycline treatment, 12z cells are loaded into the upper chamber which lacks FBS. Over 24 hours, 12z cells migrate through the Matrigel-coated membrane towards the FBS-containing media in the bottom chamber. (D) There is a trend towards greater 12z invasion after shGATA2 compared to shScramble, but this is not statistically significant. n=11, NS=not significant (PDF 282 KB) [file 43032_2024_1730_MOESM6_ESM.pdf]
