# Supplementary material for: Immunohistochemical Analysis of GATA2 Expression in Endometrium and its Relationship with Hormone Receptor Expression in Benign and Premalignant Endometrial Disorders
Source: Reprod Sci. 2024 Oct 23;31(12):3880–91. doi: 10.1007/s43032-024-01730-5 (PMC11611599; doi:10.1007/s43032-024-01730-5)
Supplement: Supplementary file 7 — Supplemental Table 1. Summary of GATA2, GATA6, ER, and PGR expression in normal endometrium, endometriosis, and endometrial atypical hyperplasia/endometrioid intraepithelial neoplasia (EAH/EIN) (DOCX 14 KB) [file 43032_2024_1730_MOESM7_ESM.docx]

**Supplemental Table 1**

| ***Marker*** | ***Cell Type*** | ***Normal Endometrium*** | ***Endometriosis*** | ***EAH/EIN*** |
| --- | --- | --- | --- | --- |
| *GATA2* | *Glandular Cells* | *High in proliferative, reduced in secretory phase* | *Reduced and decoupled from PGR* | *Preserved in glands, decoupled from PGR* |
| *GATA2* | *Stromal Cells* | *High in proliferative, reduced in secretory phase* | *Significantly reduced* | *Significantly reduced* |
| *GATA6* | *Glandular Cells* | *Low or absent* | *No significant upregulation observed* | *No significant upregulation observed* |
| *GATA6* | *Stromal Cells* | *Low or absent* | *Low or absent* | *Low or absent* |
| *ER* | *Glandular Cells* | *High in proliferative phase* | *Variable but generally maintained* | *Variable, generally maintained* |
| *ER* | *Stromal Cells* | *High in proliferative phase* | *Variable* | *Variable* |
| *PGR* | *Glandular Cells* | *High and tightly coupled with GATA2* | *Decoupled from GATA2 in some cases* | *Decoupled from GATA2* |
